# Application of In Vivo Induced Antigen Technology (IVIAT) to *Bacillus anthracis*

**DOI:** 10.1371/journal.pone.0001824

**Published:** 2008-03-19

**Authors:** Sean M. Rollins, Amanda Peppercorn, John S. Young, Melissa Drysdale, Andrea Baresch, Margaret V. Bikowski, David A. Ashford, Conrad P. Quinn, Martin Handfield, Jeffrey D. Hillman, C. Rick Lyons, Theresa M. Koehler, Stephen B. Calderwood, Edward T. Ryan

**Affiliations:** 1 Massachusetts General Hospital, Boston, Massachusetts, United States of America; 2 University of New Mexico Health Science Center, Albuquerque, New Mexico, United States of America; 3 Department of Microbiology and Molecular Genetics, The University of Texas Medical School at Houston, Houston, Texas, United States of America; 4 United States Department of Agriculture (USDA), Animal and Plant Health Inspection Service (APHIS), Sao Paulo, Brazil; 5 Centers for Disease Control and Prevention, Atlanta, Georgia, United States of America; 6 Center for Molecular Microbiology and Department of Oral Biology, College of Dentistry, University of Florida, Gainesville, Florida, United States of America; 7 Oragenics Inc, Alachua, Florida, United States of America; 8 Department of Microbiology and Molecular Genetics, Harvard Medical School, Boston, Massachusetts, United States of America; 9 Department of Medicine, Harvard Medical School, Boston, Massachusetts, United States of America; 10 Department of Immunology and Infectious Diseases, Harvard School of Public Health, Boston, Massachusetts, United States of America; Tufts University, United States of America

## Abstract

In vivo induced antigen technology (IVIAT) is an immuno-screening technique that identifies bacterial antigens expressed during infection and not during standard in vitro culturing conditions. We applied IVIAT to *Bacillus anthracis* and identified PagA, seven members of a N-acetylmuramoyl-L-alanine amidase autolysin family, three P60 family lipoproteins, two transporters, spore cortex lytic protein SleB, a penicillin binding protein, a putative prophage holin, respiratory nitrate reductase NarG, and three proteins of unknown function. Using quantitative real-time PCR comparing RNA isolated from in vitro cultured *B. anthracis* to RNA isolated from BALB/c mice infected with virulent Ames strain *B. anthracis*, we confirmed induced expression in vivo for a subset of *B. anthracis* genes identified by IVIAT, including L-alanine amidases BA3767, BA4073, and *amiA* (pXO2-42); the bacteriophage holin gene BA4074; and *pagA* (pXO1-110). The exogenous addition of two purified putative autolysins identified by IVIAT, N-acetylmuramoyl-L-alanine amidases BA0485 and BA2446, to vegetative *B. anthracis* cell suspensions induced a species-specific change in bacterial morphology and reduction in viable bacterial cells. Many of the proteins identified in our screen are predicted to affect peptidoglycan re-modeling, and our results support significant cell wall structural remodeling activity during *B. anthracis* infection. Identification of L-alanine amidases with *B. anthracis* specificity may suggest new potential therapeutic targets.

## Introduction

In vivo induced antigen technology (IVIAT) is an immuno-screening technique designed to identify immunogenic bacterial genes expressed specifically during infection [1]–[4]. We hypothesized that applying IVIAT to *Bacillus anthracis*, the cause of anthrax, could lead to increased understanding of bacterial events during infection, improved diagnostic assays, and novel therapeutic targets. IVIAT utilizes convalescent sera from patients or animals infected with a pathogen of interest [1]–[4]. Convalescent sera are pooled from several individuals and adsorbed against the cognate pathogen grown under standard laboratory culture conditions. Extensive adsorptions are performed to remove antibodies that bind bacterial antigens expressed in vitro, while retaining antibodies that recognize bacterial antigens specifically expressed during in vivo growth. Adsorbed serum is then used to probe an *Escherichia coli*-based inducible protein expression library of the pathogen. IVIAT thus provides a screen to identify proteins that generate a humoral immune response, and are displayed specifically during infection.

Thus far, IVIAT has been applied to several pathogenic bacteria including *Vibrio cholerae, Vibrio vulnificus, E. coli* O157:H7, *Salmonella enterica* serovar Typhi, *Streptococcus pyogenes* (Group A), and *Mycobacterium tuberculosis*, among others [2], [3], [5]–[9]. In these applications, IVIAT has identified pathogen genes expressed uniquely in vivo, including antigens previously established to be expressed in vivo, and newly recognized virulence factors [2], [3], [5]–[9]. Identification of up-regulated virulence genes by IVIAT has been confirmed in complementary studies, including gene microarray analysis, quantitative real time PCR (RT-PCR), signature tagged mutagenesis, and recombinase in vivo expressed technologies (RIVET), among others [9]–[11].

Certain in vitro conditions have been identified that induce expression of *B. anthracis* genes involved in pathogenesis during anthrax, including expression of toxins and capsule. For instance, growth of *B. anthracis* in Nutrient Broth Yeast extract medium (NBY) in the presence of 5–20% CO_2_ and sodium bicarbonate at 37°C (selected to mimic the physiology of blood) is associated with increased expression of protective antigen (PA) and capsule compared to that observed during growth in standard laboratory media such as Brain Heart Infusion (BHI) in the presence of air (0.03% CO_2_) at 37°C [12]. Although PA itself is non-toxic, PA binds specific host cell receptors and forms a pre-pore, which binds either edema factor (EF) or lethal factor (LF) to create edema toxin (ET) and lethal toxin (LT), respectively [13]. Anti-PA immune responses correlate with protection from anthrax, and PA is a primary immunogen in existing anthrax vaccines [14]. Full virulence of *B. anthracis* also correlates with presence of *B. anthracis* capsule, which is comprised of poly-D-glutamic acid. The presence of capsule impedes opsonophagocytosis and clearance of *B. anthracis* organisms from infected hosts [15]. Un-encapsulated strains of *B. anthracis* are markedly attenuated in both humans and animals, despite their ability to express fully functional LT and ET complexes. Beneath the capsule is a proteinaceous S-layer, composed of two paracrystalline proteins (extractable antigen 1 [EA1] and surface array protein [SAP]) that cover the bacterial cell surface [16]. Beneath the S-layer is a peptidoglycan layer comprising the cell wall.

*B. anthracis* contains two large virulence plasmids: genes encoding toxin proteins PA (*pagA*), LF (*lef*), and EF (*cya*) are on the large virulence plasmid pXO1 (182 kb; 143 ORFs), while genes encoding proteins involved in encapsulation are on the large virulence plasmid, pXO2 (96 kb; 85 ORFs). Many *B. anthracis* genes on pXO1, pXO2, and the single large chromosome have not been characterized, and it is possible that additional factors induced during infection and not under typical in vitro culture conditions contribute to *B. anthracis* pathogenesis. Identification of such gene products could improve our understanding of host-pathogen interactions during anthrax, and could assist further development of appropriate therapeutic, diagnostic, or preventative measures for anthrax.

To identify antigenic *B. anthracis* proteins displayed specifically during *B. anthracis* infection, we applied IVIAT using convalescent sera from partially immunized macaques surviving aerosol challenge with wild type *B. anthracis* Ames spores, quantified expression of identified genes in a mouse model of virulent *B. anthracis* Ames infection, and performed preliminary functional analysis on a subset of identified *B. anthracis* gene products.

## Results and Discussion

### Identification of *B. anthracis* proteins expressed in vivo by IVIAT

Using IVIAT, we immuno-screened an approximately 125,000 clone inducible *B. anthracis* expression library in *E. coli* BL21(DE3) (17,000 pXO1 clones, 7,500 pXO2 clones, and 100,000 chromosomal clones). We identified 60 immuno-reactive clones, representing 25 distinct *B. anthracis* genes or gene fragments. Of these, we constructed full length constructs and confirmed immuno-reactivity of ten (Table 1). Significant immuno-reactivity of clones containing in-frame protein-coding fragments of BXB0048 and BA2125 and lacking gene fragments of other *B. anthracis* genes was detected (these protein coding regions are not similar to any alternative *B. anthracis* proteins); however, immuno-reactivity could not be confirmed in clones containing the complete ORFs of these gene fragments, possibly suggesting inefficient expression or display of full-length products in *E. coli*. Since a number of identified seroreactive ORF products were predicted to be cell wall active amidases or hydrolases, to evaluate whether immuno-reactivity was unique to a given IVIAT-identified ORF, we also evaluated immuno-reactivity of paralogs of IVIAT-identified genes BA4073 (a predicted amidase) and pXO2-08 (a possible hydrolase and our most frequently identified ORF-product), and identified eight additional gene products with immuno-reactivity (Table 2).

**Table 1:** *B. anthracis* gene products identified by IVIAT.

Functional category	ORF	Gene product-description, function (reference)	Predicted cellular location	Gene encoded by	# of clones identified by primary screening/# of independent clones identified by primary screening	Comments
Virulence	pXO1-110 (*pagA*)	Protective antigen	Extracellular	pXO1	3/3	Known virulence factor [45], [46]
Transport	BA0314	Substrate-binding protein of an ABC transporter	Membrane associated	Chromosome	1/1	Predicted ABC metal ion transporter; contains lipoprotein domain
Transport	BA0669 (*rbsB*)	Ribose binding protein of a ribose ABC transporter	Membrane associated	Chromosome	1/1	Contains membrane-associated signal peptide sequence
Metabolism	BA2125 (*narG*)	Respiratory nitrate reductase, alpha subunit	Unknown	Chromosome	3/1	May assist organism to persist in anaerobic conditions [47].
Peptidoglycan architecture	BA2748 (*sleB*)	Spore cortex lytic protein	Unknown	Chromosome	1/1	See text
Peptidoglycan architecture	BA4073 (*plyL*)	N-acetylmuramoyl-L-alanine amidase	Extracellular, cell-associated	Chromosome	8/4	See text [17]
Peptidoglycan architecture	BA4606 (*pbpI*)	Penicillin binding protein	Extracellular, cell-associated	Chromosome	1/1	See text
Hypothetical	pXO2-08	NLP/P60 lipoprotein family	Extracellular, cell-associated	pXO2	28/11	See text
Hypothetical	BXB0048	Hypothetical protein	Unknown	pXO2	1/1	See text
Hypothetical	BA0698	Hypothetical protein	Unknown	Chromosome	1/1	See text
Hypothetical	BA4074	Prophage holin	Membrane associated	Chromosome	8/4	See text
Hypothetical	BA5474	Degenerative enterotoxin	Unknown	Chromosome	4/1	See text

**Table 2:** Immunoreactive paralogs of *B. anthracis* gene products identified by IVIAT.

Functional category	ORF	Gene product-description, function (reference)	Predicted cellular location	Gene encoded by	Paralog of IVIAT-identified gene product
Peptidoglycan architecture	BA0485	N-acetylmuramoyl-L-alanine amidase	Extracellular, cell-associated	Chromosome	BA4073
Peptidoglycan architecture	BA2446	N-acetylmuramoyl-L-alanine amidase	Extracellular, cell-associated	Chromosome	BA4073
Peptidoglycan architecture	BA2805 (*plyPH*)	N-acetylmuramoyl-L-alanine amidase; glycosyl hydrolase [48]	Extracellular, cell-associated	Chromosome	BA4073
Peptidoglycan architecture	BA3737	N-acetylmuramoyl-L-alanine amidase	Extracellular, cell-associated	Chromosome	BA4073
Peptidoglycan architecture	BA3767	N-acetylmuramoyl-L-alanine amidase	Extracellular, cell-associated	Chromosome	BA4073
Peptidoglycan architecture	pXO2-42 (*amiA*)	N-acetylmuramoyl-L-alanine amidase [49]	Extracellular, cell-associated	pXO2	BA2446, BA3737
Unknown	BA1952	NLP/P60 lipoprotein family	Extracellular, cell-associated	Chromosome	pXO2-08
Peptidoglycan architecture	BA5427 (*lytE*)	NLP/P60 lipoprotein family	Extracellular, cell-associated	Chromosome	pXO2-08

### Antigens identified by IVIAT

With respect to novel *B. anthracis* antigens identified by IVIAT, we were most intrigued by the observation that a number of identified proteins were predicted to be involved in cell wall/peptidoglycan synthesis or remodeling. In particular, our application of IVIAT to *B. anthracis* identified a number of L-alanine amidases, a family of enzymes that cleaves an amide bond between N-acetylmuramic acid and L-alanine in bacterial cell wall peptidoglycan [17]. Interestingly, L-alanine amidases are also present in a number of phages with lytic capabilities specific against *B. anthracis*. For instance, within the NCBI database, the highest similarity protein to L-alanine amidase BA4073 identified by IVIAT is the *B. anthracis* specific gamma phage lysin, PlyG, with 82% identity over the full length of both proteins, and PlyG has been demonstrated to function as a species-specific *B. anthracis* lysin [18]. We also identified prophage holin BA4074 by IVIAT. Holins act upon bacterial cytoplasmic membranes and, in conjunction with an endolysin, promote lysis of infected bacteria [19]. Akin to our identification of amidase BA4073 and prophage holin BA4074 by IVIAT, we similarly identified amidase BA3767, which is also in a potential operon arrangement with a prophage holin, BA3768.

Using IVIAT, we also identified spore cortex lytic protein (BA2748), a protein with marked similarity to characterized *Bacillus cereus* and *Bacillus subtilis* SleB proteins. The latter function as cell wall hydrolases and are required for complete spore-cortex peptidoglycan hydrolysis during germination [20]–[22]. BA2748 also contains a putative peptidoglycan-binding domain found in a variety of enzymes involved in bacterial cell wall degradation, and expression is induced late during sporulation in *B. anthracis* [23], although the enzymatic activity in *B. subtilis* and *B. cereus* occurs during germination [20], [21]. We were also intrigued by our identification of BA4606 that annotates as a penicillin-binding protein. Penicillin-binding proteins are a broad group of membrane-associated macromolecules involved in peptidoglycan biosynthesis, cell wall development, and cell division. BA4606 contains an FtsI cell division protein/penicillin-binding protein 2 domain, and the FtsI penicillin-binding protein family is required for septal peptidoglycan formation during cell division [24]. BA4606 is also 45% identical to *B. subtilis* hypothetical protein YrrR (also referred to as PBP4b, *pbpI*) over the full length of both proteins. *B. subtilis* YrrR is a mother-cell specific penicillin-binding protein expressed during sporulation [25]. The FtsI domain contains a penicillin-binding protein transpeptidase domain, which putatively cross-links peptidoglycan chains to form a rigid cell wall structure.

We also identified four hypothetical proteins. The most frequently identified gene product identified by IVIAT was ORF pXO2-08. pXO2-08 is predicted to be an extracellular protein [26], and contains a P60 family lipoprotein domain. pXO2-08 also has sequence similarity to a murein hydrolase domain from *Listeria monocytogenes* P60 protein (alternatively referred to as *iap*-invasion associated protein) [27]. Several proteins containing a P60 domain have been implicated in virulence, including two P60 domain proteins in *Mycobacterium marinum* involved in invasion and intracellular survival within macrophages [28], and a *L. monocytogenes* mutant in P60 is severely attenuated, suggesting a potential role in virulence [29]. Although the function of the P60 lipoprotein domain is not completely understood, it is conserved through a variety of bacterial lineages, and some members of this family have been shown to act as murein hydrolases that cleave peptide linkages within peptidoglycan [30]. Immuno-screening of pXO2-08 paralog ORF clones identified two additional *B. anthracis* serologically reactive paralogs in the P60 lipoprotein family: BA1952 and BA5427 (*lytE;* putative endopeptidase). BA1952 and BA5427 have been demonstrated to be antigenic secreted proteins, and both contain P60 and S-layer homology (SLH) domains [31]–[33]. SLH domains are commonly associated with cell surface proteins and non-covalently anchor proteins to the cell wall polysaccharide, suggesting cell surface localization [34]. BA5427 is homologous to *B. subtilis lytE*, and LytE (also referred to as CwlF) has been shown to function as a cell wall lysin that affects bacterial cell separation in *B. subtilis* [35]. Our application of IVIAT to *B. anthracis* also identified BA5474, encoding a pseudogene that has sequence similarity to several genes encoding N-acetylmuramoyl-L-alanine amidases and an enterotoxin in *B. cereus* [36].

### RT-PCR quantification of in vivo expression of IVIAT-identified and paralog *B. anthracis* genes during wild type *B. anthracis* infection

**Figure 1:**
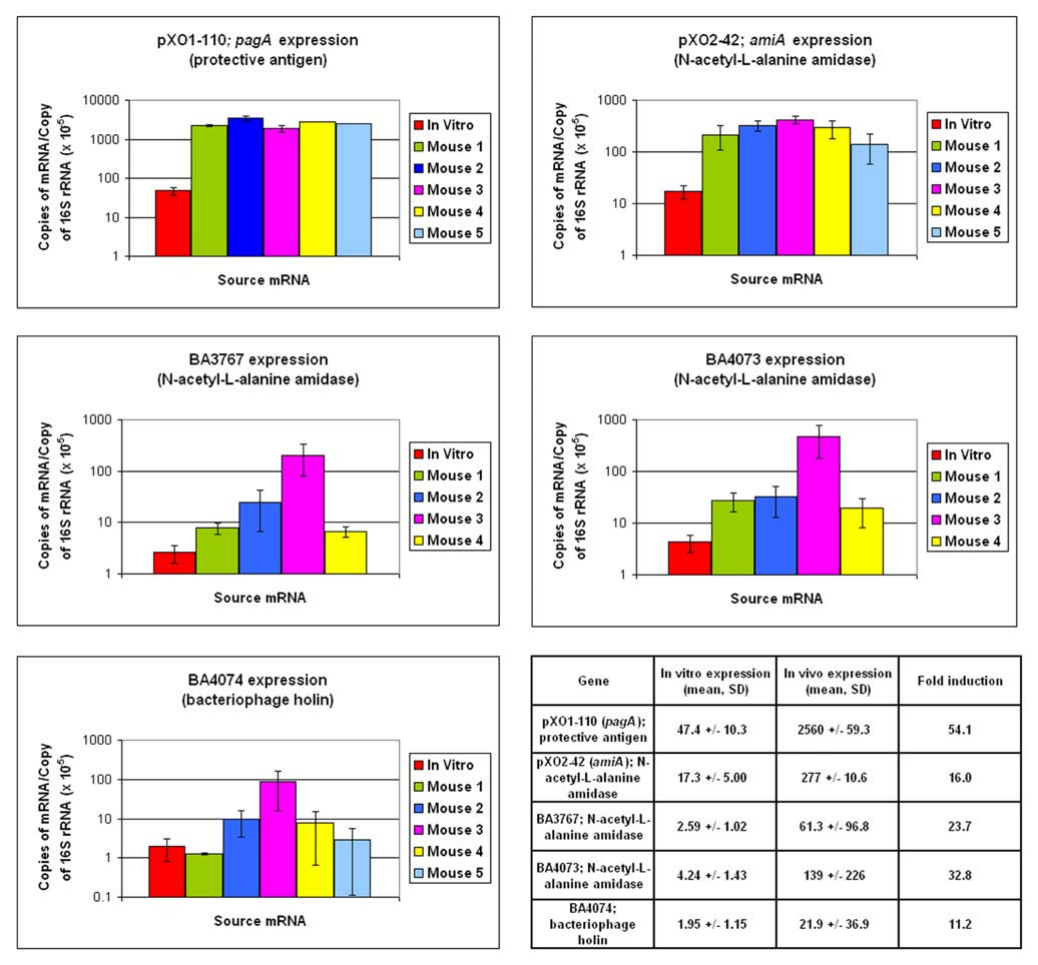
mRNA expression profiles during in vitro and in vivo growth of IVIAT-identified and paralog/similar *B. anthracis* genes. Quantitative RT-PCR was performed on RNA recovered from in vitro grown *B. anthracis* (Ames strain cells grown in BHI and air; late-log phase) and compared to RNA recovered from mice infected with vegetative Ames strain *B. anthracis* via intravenous injection. RNA from mice was isolated from spleens 18 hr post-infection. Transcript numbers on the Y-axis are normalized against 16S rRNA. Displayed are gene profiles of *B. anthracis* genes up-regulated in vivo compared to in vitro. In bar graphs, expression levels represent mean values; error bars represent standard deviations (SD). In summary table, expression given as mRNA transcript copies (× 10^−5^) per copy of 16S rRNA.

To further assess in vivo expression of IVIAT-identified ORF products, we measured mRNA levels of antigens identified by IVIAT. Comparing total bacterial RNA recovered from blood, spleen, and lung of mice 6, 12, or 18 hours after inoculation of vegetative *B. anthracis* Ames, we detected the highest quantity of bacterial RNA in spleen specimens recovered 18 hours after infection. Based on rRNA 16S concentrations, spleen specimens contained bacterial to mouse RNA in ratios ranging from 1∶100 to 1∶2,000. Comparing RNA recovered from spleen samples to RNA recovered from in vitro cultured organisms grown under the same conditions used to adsorb sera, we detected up-regulation of five *B. anthracis* IVIAT-identified genes or paralogs/similar proteins in in vivo samples using RT-PCR (Figure 1): *pagA*; the alanine-amidase genes, BA3767, BA4073, and *amiA* (pXO2-42); and the bacteriophage holin gene BA4074. Within our assay, *pagA* mRNA was present at approximately 54-fold higher levels in in vivo samples than in vitro samples. mRNA of L-alanine amidases BA4073, BA3767, *amiA* (pXO2-42), as well as prophage holin BA4074 were also elevated in in vivo samples compared to in vitro samples (33-fold, 24-fold, 16-fold, and 11-fold higher, respectively).

Our identification of PA by IVIAT and our confirmation of up-regulation of *pagA* mRNA in vivo confirms the validity of our approach since PA is preferentially expressed in vivo and is immunogenic [12], [14]. Of note, our inducible *B. anthracis* library was constructed using *B. anthracis* strains that did not encode LF or EF, and we would, therefore, not expect our screens to have identified these virulence factors. Our documentation of induced expression of mRNA in vivo of only a subset of genes identified by IVIAT (and not the complete set) is also not unexpected, since mRNA quantity is only one of a number of variables that may alter expression or display of a given protein. Indeed, separate proteomic based analysis has recently demonstrated increased protein expression of two additional *B. anthracis* gene products that we have identified by IVIAT, lipoproteins BA1952 and BA5427, when *B. anthracis* are grown in high versus low CO_2_ concentrations (the former conditions thought to mimic the in vivo environment) [31]. In addition, a number of IVIAT identified antigens (BA0314, BA0485, BA1952, BA2805, BA2748, BA3737, BA5427, pXO2-08 and pXO2-42) were also previously identified by Gat et al. using a bioinformatics screening strategy to select *B. anthracis* ORFs for use in an in vitro transcription/translation assay followed by immunoprecipitation using sera from *B. anthracis*-infected animals [37]. Identification of these ORF products by both our application of IVIAT to *B. anthracis*, and by Gat et al.'s screening system, support identified ORF-product immuno-reactivity in vivo.

### Species-specific effects of N-acetyl-L-alanine amidases BA0485 and BA2446 on *B. anthracis*

**Figure 2:**
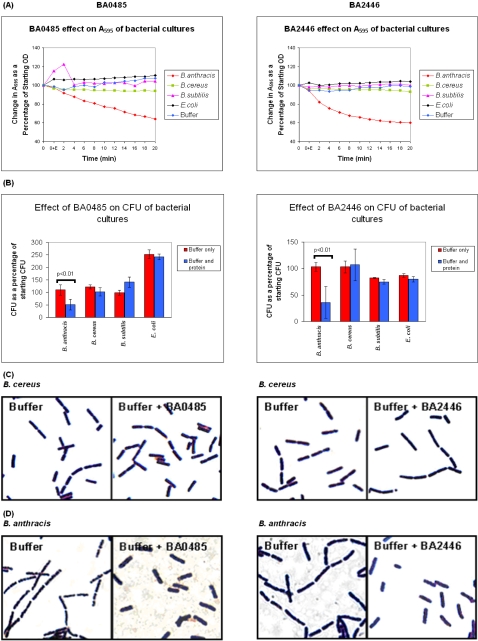
*B. anthracis* species-specific autolysis effects following addition of exogenous putative autolysins N-acetylmuramoyl-L-alanine amidases BA0485 and BA2446: (A) reduction in A_595_ optical density, (B) reduction in colony forming units, and (C, D) morphological changes of *B. cereus* (C) or *B. anthracis* (D). *B. anthracis* (Sterne strain), *B. cereus* (ATCC 14579), *B. subtilis* (168), and *E. coli* (DH5α) vegetative cells were resuspended in 20 mM sodium phosphate buffer containing BA0485 and BA2446 (final concentration 2 uM) or buffer alone (BA0485, pH 7.0; BA2446, pH 8.7). (A) A_595_ readings were recorded every 2 min. and reported as a percentage of starting A_595_. Buffer-only controls were not different for cultures containing *B. cereus, B. subtilis,* or *E. coli*. (B) Dilutions of cells were plated prior to addition of BA0485 and BA2446 proteins and then again following 20 min of incubation with buffer alone or buffer containing exogenous BA0485 or BA2446, and CFU are reported as percentage of the starting CFU. (C, D) BA0485, BA2446, or buffer alone were added to cell suspensions of *B. anthracis*, *B. cereus, B. subtilis,* and *E. coli*, and incubated for 20 min. Bacterial morphological changes were only evident following protein addition to *B. anthracis* (D); representative *B. cereus* samples are shown (C). Gram stains were performed and all images were captured at 1000×.

We were intrigued that a number of the *B. anthracis* proteins identified in our analysis were predicted to be involved in cell wall-peptidoglycan synthesis or remodeling, and that a number were homologous to known lysins. In order to investigate this further, we focused our efforts on the best characterized family of identified proteins, the L-alanine amidases. We were able to purify recombinant versions of two identified amidases, BA0485 and BA2446. Upon addition of exogenous L-alanine amidases BA0485 (buffered at pH 7.0) or BA2446 (buffered at pH 8.7), the OD_595_ of a cell suspension made from exponential phase organisms fell specifically for *B. anthracis*, but not for *B. cereus*, *B. subtilis*, or *E. coli* DH5α (Figure 2A). This drop in OD_595_ corresponded to a 50% reduction in CFU for *B. anthracis* grown in the presence of BA0485 or BA2446, and this reduction in CFU was not noted in cultures containing BA0485 or BA2446 and *B. cereus*, *B. subtilis*, *E. coli*, or buffer alone (Figure 2B). *B. anthracis* vegetative bacteria cultured in the presence of exogenous BA0485 or BA2446 also had decreased chain length compared to *B. anthracis* cultured in buffer alone (BA0485: 1.06 [mean] +/−0.01 [standard error of mean] versus 2.17 +/−0.08, p<0.001; BA2446: 1.14 +/−0.02 versus 2.43 +/−0.07, p<0.001; Figure 2C–D), as well as a more swollen appearance. The exogenous addition of BA0485 or BA2446 to cultures of *B. cereus*, *B. subtilis,* or *E. coli* had no measurable effect on bacterial morphology, chain length, or viability. Although our exogenous addition of these proteins to in vitro grown cultures may not represent their in vivo location or activity, our data strongly suggest an effect of these proteins on *B. anthracis* cell wall integrity, a focus of on-going work in our laboratory. We also noted an optimal pH requirement (pH = 8.7) for the BA2446 product in vitro, and although similar pH dependence has previously been noted for autolysins specific for *B. subtilis* [38], the physiological significance of this observation in vivo is currently unclear.

Although our application of IVIAT to *B. anthracis* has identified a number of genes warranting additional evaluation, our study has a number of limitations. (1) IVIAT only identifies immuno-reactive proteins (not carbohydrates or lipids). (2) Our inducible protein library was undoubtedly only partial due to the *Sau*3A partial digestion used to generate library clones, the effect of codon bias on protein expression, and difficulties expressing all antigens (especially membrane associated proteins) equivalently in *E. coli* based expression libraries. (3) Immuno-reactivity of IVIAT-identified antigens is a marker of expression of proteins in vivo, and does not predict protective humoral or cellular immune responses. (4) Immuno-reactivity of proteins may also reflect immune responses to related antigens, although we have attempted to identify cross-reactivity in these experiments by also analyzing related proteins. (5) Due to genetic cross-talk between the virulence plasmids and the chromosome of *B. anthracis* [39], our use in the adsorption protocol of attenuated *B. anthracis* strains that did not contain the complete genome in a single strain may have misrepresented potential gene expression in vitro. (6) Our use of convalescent phase sera from macaques partially immunized against anthrax and then surviving inhalational challenge with virulent *B. anthracis*, may not exactly mirror events that occur during natural infection in un-immunized humans or animals, and anti-PA responses in macaques may not correlate with overall anti-*B. anthracis* immunoreactivity. (7) Our use of the intravenously-challenged mouse model to measure bacterial gene expression in vivo may not exactly reflect events that occur during natural infection (since the mouse model involves neither spore germination nor formation); however, using this model, we were still able to detect induction of a number of IVIAT identified genes or paralogs in vivo.

Despite these limitations, our results indicate host antibody responses and increased bacterial production during infection of several proteins, including a subset of seven L-alanine amidases, five proteins containing lipoprotein domains, a prophage holin, spore cortex lytic protein SleB, and a penicillin-binding protein that may be involved in cell wall/peptidoglycan synthesis, degradation, and/or remodeling. Fisher et al have recently shown that a *B. anthracis* locus (*dltABCD*) involved in cell wall L-alanine esterification is involved in endospore remodeling and is required for full virulence of *B. anthracis* in vivo [40]. Our results further support prominent cell wall structural remodeling activity during anthrax, may suggest new diagnostic targets to detect *B. anthracis* infection, and our identification of predicted L-alanine amidases with *B. anthracis* specificity may suggest new potential alternative therapeutic approaches against this important bacterial pathogen.

## Materials and Methods

### Bacterial strains and conditions

*Bacillus* species and strains used in these experiments are described in Table 3. *E. coli* BL21(DE3) and BL21(DE3) Codon Plus-RIL strains (Stratagene, La Jolla, CA) were grown in Luria Bertani (LB) media supplemented with appropriate antibiotics.

**Table 3:** *Bacillus* strains used in this study.

Species	Strain	Relevant Characteristics	Source/Reference
*Bacillus anthracis*	7702	Sterne strain; pXO1+, pXO2-	[50], [51]
	6602	Pasteur strain; pXO1-, pXO2+	ATCC
	9131	Sterne strain 7702 derivative cured of pXO1; pXO1-, pXO2-	[52]
	9131(pXO2)	9131 containing pXO2 from 6602; pXO1-, pXO2+	[53]
	RP42	Sterne strain 7702 derivative; pXO1+ (Δ*lef*, Δ*cya*), pXO2-	[54]
	Ames	Virulent wild type; pXO1+, pXO2+	US Army Medical Research Institute of Infectious Diseases, Frederick, MD
*Bacillus cereus*	14579	Wild type strain	ATCC #14579
*Bacillus subtilis*	168 [EMG 51]	Wild type strain	ATCC #23857

### Construction of an inducible *B. anthracis* genomic DNA expression library

We constructed a BL21(DE3)-based inducible expression library including *Sau3*A derived 0.5–1.5 kb *B. anthracis* genomic fragments in pET30abc using DNA from *B. anthracis* RP42, 9131, and 9131(pXO2) and previously described techniques [3], [6], [41], [42].

### Adsorbing convalescent macaque serum to *B. anthracis* grown in vitro

Healthy rhesus macaques (*Macaca mulatta*) in the weight range of 2.6 kg to 4.5 kg were quarantined for a minimum of 6 weeks prior to the study start. We obtained convalescent sera from nine macaques that had been immunized with three doses (week 0, 4, 26) of a 1∶20 or 1∶40 dilution of anthrax vaccine adsorbed (AVA), and survived aerosol challenge with 20–422 LD_50_ equivalents (1.1×10^6^–23.2×10^6^ CFUs) of *B. anthracis* Ames strain at week 52. Sera were harvested on days 14 and 30 post-challenge. *B. anthracis* Ames infection of macaques was confirmed by seroconversion to anti-PA IgG following aerosol exposure. We pooled sera containing the highest anti-PA antibody levels from each surviving macaque. We removed antibodies binding in vitro expressed proteins by six successive direct adsorptions with concentrated whole *B. anthracis* cells (10^11^ CFUs/ml RP42 and 9131 [pXO2] mixed in equal volumes) grown in vitro in BHI broth in air at 37°C until mid/late log phase [3], [6]. We further adsorbed pooled sera with both native and heat-denatured cell lysates of mid/late-log phase cultures of *B. anthracis* RP42 and *B. anthracis* 9131(pXO2) (1∶1) and *E. coli* BL21 (DE3), as previously described [3].

### Primary screening of the protein expression library, identification of inserts, and prediction of function of antigens identified by IVIAT

For primary screening, we followed a modification of the method of Hang et al [6]. Briefly, we plated aliquots of the protein expression library on LB agar-kanamycin plates and incubated plates for approximately 10 hours at 37°C, and then used nitrocellulose disks to replica plate colonies onto LB agar-kanamycin plates containing 1 mM isopropyl-beta-D-thiogalactoside (IPTG), and incubated plates at 37°C for 3–4 hours. To expose induced antigens, we lysed bacterial colonies by saturating disks with chloroform for ten seconds and air-drying. Following washing and blocking of disks, we incubated membranes with adsorbed sera at a 1∶6,000 dilution in PBS-Tween at 4°C overnight with mild agitation, and detected immuno-reactive clones using horse-radish peroxidase-conjugated goat anti-monkey IgG (Kirkegaard and Perry Laboratories, Gaithersburg, MD) at a 1∶20,000 dilution in PBS-Tween and an ECL chemiluminesence kit with Hyperfilm ECL (Amersham, Pascataway, NJ). We recovered plasmid DNA from positive clones and sequenced *B. anthracis* DNA inserts. We surveyed insert DNA for open reading frames, regulatory sequences, and signal sequences. Functional classification was based, when available, on published studies of identified proteins in *B. anthracis*. When no published functional studies of the *B. anthracis* genes were available, protein functional classification was based on annotation from the *B. anthracis* Ames chromosome (NCBI accession # NC_003997), pXO1 (NCBI accession # NC_001496), pXO2 (NCBI accession # AF188935), and predictive models using BLASTP protein alignment analysis and a Conserved Domain Database (CDD) domain search (http://www.ncbi.nlm.nih.gov/BLAST/). Cellular localization of antigens identified by IVIAT was predicted using PSORTb version 2.0.4. [26]; http://www.psort.org/psortb/).

### Immuno-screening of individual gene products, paralogs, and similar proteins

We used PCR to generate the entire predicted *B. anthracis* ORF contained within each insert and cloned these into pET30abc; we confirmed reactive ORF products with serological immuno-screening, as above. We also evaluated serological reactivity of bioinformatically selected paralog and similar protein ORF clones. Specifically, for ORF pXO2-08, we evaluated paralogs BA1952, BA2849, and BA5427; and for ORF BA4073, we evaluated structurally or functionally related amidases including BA0485, BA0872, BA2446, BA2805, BA3737, BA3767, and pXO2-42 (*amiA*).

### Assessment of in vivo expression of IVIAT-identified, paralog and similar *B. anthracis* genes

To assess in vivo expression of *B. anthracis* genes identified by IVIAT, we inoculated tail veins of BALB/c mice (Harlan Laboratories, Indianapolis, IN) with 200-LD_50_ of *B. anthracis* Ames vegetative cells growing in log phase under conditions associated with expression of virulence factors and capsule [43]; we inoculated control mice with PBS. We collected blood, lungs, and spleens of mice sacrificed 6, 12, or 18 hours following inoculation, immediately homogenizing tissues and adding RNAwiz reagent (Ambion; Austin, TX) to recovered samples. We extracted RNA using an Ambion RiboPure-Bacteria kit (Austin, TX), following manufacturer's specifications. To measure *B. anthracis* gene expression in vitro, we similarly recovered RNA from Ames strain *B. anthracis* vegetative cells grown in BHI and air to mid/late log phase. We quantified RNA preparations spectrophotometerically, removed residual DNA using an Ambion DNAfree kit, and generated cDNAs using an Ambion Retroscript kit.

We designed primers (Table 4) using Beacon Design (Premier Biosoft; Palo Alto, CA), www.primer3.com, and Blast software. We performed RT-PCR analysis on collected mouse and in vitro grown samples using iQ SYBR Green Supermix reagent (Bio-Rad; Hercules, CA) and a MJ Research Chromo4 thermocycler and MJ Opticon Monitor software version 3.1 (Bio-Rad; Hercules, CA). After an initial denaturation at 95°C for 3 min, the RT-PCR cycle was as follows: denaturation at 94°C for 30 sec, extension at 58°C for 30 sec, extension at 72°C for 1 min, followed by a plate read. The cycle was repeated for a total of 40 cycles. C(t) values were set in the low/linear portion of product curves. We quantified gene copy numbers against pET30-based plasmids containing the gene of interest; we calculated control gene copy numbers using plasmid size, and spectrophotometerically determined A_260_ readings. We normalized gene copy numbers against copies of 16S rRNA cDNA [44]. Controls included samples lacking reverse transcriptase, and a no template control to provide a reference baseline reaction. We assessed singularity of product species and size by Metaphor gel (Cambrex Bio-Science; Rockland, ME) and melting curve analysis.

**Table 4:** Primers for RT-PCR quantification of cDNA isolated from Ames strain *B. anthracis* recovered from infected mice or from in vitro grown *B. anthracis*.

Gene	Upstream primer	Downstream primer
16S	TAGGTGGCAAGCGTTATC	GGTGTTCCTCCATATCTCTAC
BA0314	TAGGGGTTTCAGCATTAAG	GTGTAATCAGAGAACTCAAC
BA0669	TGTTCAATGGAACCACCAGA	ATCAATTCAATGCCGCTTTC
BA0698	GCAGAAATACTCGTTGCCATT	GCTAGCAGTTCCGAGAAGGA
BA2125	GTGGTAAAGGGGGATTCGTT	GCCTGCAGCATGACTTAACA
BA2748	ATCGGGCGAAGATGTTATTG	ACGAGCATTTGCTTCGTTTT
BA4073	ATTGCCCACATAGAATGC	TATAATCGCCTTACCTGTC
BA4074	GGTGGTGCTTTTTCTTTTGG	GTTAAAGCCTGTGGGAGTGG
BA4606	TTAATTGGCAATGTGGGTGA	GCCGGTCCACTTGATACAAT
BA5474	GGCGAATTCGTATCTGGTGT	TGAGAAGTGCTAGGGCCTGT
pXO1-110 (*pagA*)	GCATGCGTCGTTCTTTGATA	CCATTGTTTCAGCCCAAGTT
pXO2-08	TGGAACTTCAGGGCAAATTC	GCCGTTTTATACGCCCAATA
BXB0048	TTTGGCTGTCAGTTTTGCTG	TCACTCCCCATTTCCACATA
**Primers for selected paralogs/homologs**		
**Paralog/similar proteins of pXO2-08**		
BA1952	ATCTTCAATCGCTGGATTCG	TCCAGCAACTGTTTGACGAG
BA5427	ACAAGAAGCTGCAGCACAAA	CTGGTGTCGGTTCTGGTTTT
**Paralog/similar proteins of BA4073**		
pXO2-42 (*amiA*)	TACCAAACTGGGCACAACAA	GCATCCTGAAATGACGGTTT
BA0485	ACAGTTTGGGTGGCTGATGT	CGCCATACCGAAAGGTGTAT
BA2446	AAATCGTGGTGCAAAGGAAC	TTTAGATGGCGGAGTTGTCC
BA2805	GCCGCTTTCTTGGTTTACTG	AACGCTCCCATAACATCTGG
BA3737	GACAGGTATTGCGTATATTGAGG	CCACTGATTGCCACCAA

### Generation of predicted amidases BA0485 and BA2446 as histidine-tagged fusion proteins

A number of antigens identified using IVIAT were predicted to be amidases that could be involved in cell wall synthesis or remodeling. We, therefore, attempted to produce these amidases as recombinant proteins in sufficient quantities to assess physiologic effects. We were able to successfully express two such proteins. Specifically, we expressed BA0485 and BA2446 proteins containing a carboxyl-terminal hex-histidine tag from pET30-based vectors in *E. coli* BL21(DE3) Codon Plus-RIL induced with 1 mM IPTG. We recovered these proteins using affinity chromatography, and assessed protein concentration and purity using SDS-PAGE analysis and Coomasie Plus Reagent (Pierce, Rockford, IL).

### Functional activity of exogenous BA0485 and BA2446 recombinant amidase proteins on vegetative cell suspensions of *Bacillus* sp. and *E. coli*

To begin to determine the effect of identified amidases on *B. anthracis* cell wall integrity, we incubated a 1∶40 dilution in LB broth of overnight cultures of *B. anthracis* Sterne strain, *B. cereus* ATCC 14579, *B. subtilis* 168, and *E. coli* DH5α for 2–2.5 hrs in an environmental shaker at 37°C. We centrifuged cultures, resuspended bacteria to an A_595_ of 0.4 in 20 mM sodium phosphate buffer (pH 4.7, 7.0, and 8.7), added purified BA0485 or BA2446 protein to a final concentration of 2 uM to cell suspensions, and measured A_595_ every 2 min. Control samples contained buffer lacking recombinant protein. We quantified viable bacterial CFUs using LB agar plates prior to addition of BA0485 or BA2446 protein, and then again after 20 min of incubation. We assessed cellular morphology via Gram stain and light microscopy, and calculated mean vegetative cell chain length based on ten fields of view.

### Biosafety

All work described in this experiment involving *B. anthracis* strains listed in the Centers for Disease Control and Prevention Select Agent Program was performed in fully-certified BL2 and BL3 facilities, was fully approved, and met all institutional and local, state, and federal governmental regulations. Procedures that required BL3 animal work involving macaques were performed through the Centers for Disease Control and Prevention; procedures that required BL3 animal work involving mice were performed through the University of New Mexico Health Science Center, Albuquerque, NM.
